# Real-World Outcome of Treatment with Single-Agent Ibrutinib in Italian Patients with Chronic Lymphocytic Leukemia: Final Results of the EVIdeNCE Study

**DOI:** 10.3390/cancers16061228

**Published:** 2024-03-20

**Authors:** Francesca Romana Mauro, Potito Rosario Scalzulli, Lydia Scarfò, Carla Minoia, Roberta Murru, Paolo Sportoletti, Ferdinando Frigeri, Francesco Albano, Nicola Di Renzo, Alessandro Sanna, Luca Laurenti, Massimo Massaia, Ramona Cassin, Marta Coscia, Caterina Patti, Elsa Pennese, Agostino Tafuri, Annalisa Chiarenza, Piero Galieni, Omar Perbellini, Carmine Selleri, Catello Califano, Felicetto Ferrara, Antonio Cuneo, Marco Murineddu, Gaetano Palumbo, Ilaria Scortechini, Alessandra Tedeschi, Livio Trentin, Marzia Varettoni, Fabrizio Pane, Anna Marina Liberati, Francesco Merli, Lucia Morello, Gerardo Musuraca, Monica Tani, Adalberto Ibatici, Giulia Regazzoni, Michele Di Candia, Maria Palma, Danilo Arienti, Stefano Molica

**Affiliations:** 1 Ematologia, Sapienza Università di Roma, 00185 Roma, Italy; 2 Ospedale “Casa Sollievo della Sofferenza”, 71013 San Giovanni Rotondo, Italy; 3 Università Vita-Salute San Raffaele, 20132 Milano, Italy; 4 IRCCS Ospedale San Raffaele, 20132 Milano, Italy; 5 Hematology Unit, IRCCS Istituto Tumori “Giovanni Paolo II”, 70124 Bari, Italy; 6 Hematology and Stem Cell Transplantation Unit, Ospedale Oncologico A. Businco, ARNAS G. Brotzu, 09134 Cagliari, Italy; 7 Sezione di Ematologia, Università degli Studi di Perugia, 06123 Perugia, Italy; 8 AORN “Sant’Anna e San Sebastiano”, 81100 Caserta, Italy; 9 Hematology and Stem Cell Transplantation Unit, Department of Precision and Regenerative Medicine and Ionian Area (DiMePRe-J), University of Bari “Aldo Moro”, 70121 Bari, Italy; 10 UOC Ematologia e Trapianto di Cellule Staminali-PO “Vito Fazzi” ASL Lecce, 73100 Lecce, Italy; 11 AOU Careggi, 50121 Firenze, Italy; 12 Policlinico A. Gemelli, 00168 Roma, Italy; 13 SC Ematologia-AO S. Croce e Carle, 12100 Cuneo, Italy; 14 Dipartimento di Biotecnologie Molecolari e Scienze per la Salute, Università degli Studi di Torino, 10124 Torino, Italy; 15 Fondazione IRCCS Cà Granda Ospedale Maggiore Policlinico of Milan, 20122 Milano, Italy; 16 SC Ematologia U, AOU Città della Salute e della Scienza di Torino, 10126 Torino, Italy; 17 UOC Oncoematologia, Ospedali Riuniti Villa Sofia-Cervello, 90146 Palermo, Italy; 18 UOC Ematologia Clinica, ASL Pescara, 65124 Pescara, Italy; 19 Ematologia, AOU Sant’Andrea, Sapienza Università di Roma, 00185 Roma, Italy; 20 UOC Ematologia, AOU Policlinico G.Rodolico-San Marco, 95123 Catania, Italy; 21 UOC Ematologia e Terapia Cellulare, Ospedale Mazzoni, 63100 Ascoli Piceno, Italy; 22 UOC Ematologia, Azienda ULSS 8 Berica, 36100 Vicenza, Italy; 23 Hematology and Bone Marrow Transplant Center, Department of Medicine and Surgery, University of Salerno, 84081 Salerno, Italy; 24 UOC Ematologia, PO Andrea Tortora-Pagani, 84016 Pagani, Italy; 25 AORN Antonio Cardarelli, 80131 Napoli, Italy; felicettoferrara@katamail.com; 26 Department of Medical Science, Università degli Studi di Ferrara, 44121 Ferrara, Italy; 27 Ematologia, Ospedale San Francesco ASL Nuoro, 08100 Nuoro, Italy; 28 SC Ematologia, AOU Policlinico Foggia, 71122 Foggia, Italy; 29 Azienda Ospedaliero Universitaria delle Marche, 60126 Ancona, Italy; 30 Divisione di Ematologia, ASST GOM Niguarda, 20162 Milano, Italy; 31 Dipartimento di Medicina, UOC Ematologia, Università di Padova, 35122 Padova, Italy; 32 Divisione di Ematologia, Fondazione IRCCS Policlinico San Matteo, 27100 Pavia, Italy; 33 Division of Hematology, Department of Clinical Medicine and Surgery, Università di Napoli Federico II, 80131 Napoli, Italy; 34 AO Santa Maria, Università degli Studi Di Perugia, 05100 Terni, Italy; 35 Ematologia AUSL-IRCCS Reggio Emilia, 42123 Reggio Emilia, Italy; 36 IRCCS Humanitas Research Hospital-Humanitas Cancer Center, 20089 Rozzano, Italy; 37 IRCCS Istituto Romagnolo per lo Studio dei Tumori (IRST) “Dino Amadori”, 47014 Meldola, Italy; 38 Haematology Unit, Ospedale S. Maria delle Croci, 48121 Ravenna, Italy; 39 IRCCS Ospedale Policlinico San Martino, 16132 Genova, Italy; 40 Johnson & Johnson Innovative Medicine, 20126 Milano, Italy; 41 Dipartimento Onco-Ematologico, Azienda Ospedaliera Pugliese-Ciaccio, 88100 Catanzaro, Italy; 42 Queens Centre for Oncology and Haematology, Castle Hill Hospital, Hull University NHS Trust, Hull HU16 5JQ, UK

**Keywords:** chronic lymphocytic leukemia, ibrutinib, real-world evidence, retention, clinical outcomes, effectiveness

## Abstract

**Simple Summary:**

In clinical trials, ibrutinib was found to be effective and well-tolerated in patients with chronic lymphocytic leukemia (CLL). To confirm these findings, data on unselected patients treated in clinical practice are necessary. The aim of our observational, prospective Italian cohort study was to describe the real-world persistence rate, patterns of use, and clinical outcomes in patients with CLL treated with single-agent ibrutinib across various treatment lines. We found that, despite the high burden of patient comorbidities and unfavorable genetic features, the majority of patients (217/309, 70%), especially those treated in first line (75%), continued ibrutinib treatment for ≥2 years. The most common reasons for treatment discontinuation were adverse events, primarily infections. We reported positive clinical and survival outcomes, especially in the first-line cohort, and a safety profile consistent with clinical trial data. Our data suggest that ibrutinib is a valuable option for both treatment-naïve and previously treated patients with CLL.

**Abstract:**

Real-world data in clinical practice are needed to confirm the efficacy and safety that ibrutinib has demonstrated in clinical trials of patients with chronic lymphocytic leukemia (CLL). We described the real-world persistence rate, patterns of use, and clinical outcomes in 309 patients with CLL receiving single-agent ibrutinib in first line (1L, *n* = 118), 2L (*n* = 127) and ≥3L (*n* = 64) in the prospective, real-world, Italian EVIdeNCE study. After a median follow-up of 23.9 months, 29.8% of patients discontinued ibrutinib (1L: 24.6%, 2L: 29.9%, ≥3L: 39.1%), mainly owing to adverse events (AEs)/toxicity (14.2%). The most common AEs leading to discontinuation were infections (1L, ≥3L) and cardiac events (2L). The 2-year retention rate was 70.2% in the whole cohort (1L: 75.4%, 2L: 70.1%, ≥3L: 60.9%). The 2-year PFS and OS were, respectively, 85.4% and 91.7% in 1L, 80.0% and 86.2% in 2L, and 70.1% and 80.0% in ≥3L. Cardiovascular conditions did not impact patients’ clinical outcomes. The most common AEs were infections (30.7%), bleeding (12.9%), fatigue (10.0%), and neutropenia (9.7%), while grade 3–4 atrial fibrillation occurred in 3.9% of patients. No new safety signals were detected. These results strongly support ibrutinib as a valuable treatment option for CLL.

## 1. Introduction

Chronic lymphocytic leukemia (CLL), the most frequent adult leukemia in developed countries, is characterized by the clonal expansion of B lymphocytes in the blood, bone marrow, and lymph nodes [1,2]. The incidence rates of CLL in Europe and the USA range between 4 and 5 cases per 100,000 persons-year. CLL typically occurs in older individuals (median age at diagnosis: 72 years) and is more common in men than women [3].

CLL exhibits an extremely variable clinical course [1]. While some patients remain asymptomatic for decades, with a nearly normal life expectancy, others experience disease progression requiring therapeutic intervention and could eventually become refractory to therapy. The transformation of CLL into very aggressive diffuse large B-cell lymphoma or Hodgkin’s lymphoma (Richter’s transformation) occurs in about 5–10% of CLL patients [4].

For decades, chemoimmunotherapy using anti-CD20 monoclonal antibodies has been the standard of care for CLL treatment. However, the recent emergence of targeted therapies has dramatically transformed the treatment landscape, greatly improving progression-free survival (PFS) and overall survival (OS) in patients with CLL [5]. These targets agents include covalent Bruton’s tyrosine kinase (BTK) inhibitors, such as ibrutinib; acalabrutinib and zanubrutinib phosphatidylinositol-3-kinase inhibitors, such as idelalisib; and B-cell lymphoma-2 (BCL-2) inhibitors, such as venetoclax. In particular, ibrutinib, a first-in-class, once-daily, oral irreversible covalent BTK inhibitor, inhibits B-cell antigen receptor (BCR) signaling pathways in malignant B cells, promoting egress of malignant B cells from lymph nodes; and prevents homing of these cells to tissues in patients with B-cell malignancies, without having clinically adverse effects on levels of normal B cells [6].

Multiple randomized clinical trials (RCTs) [7,8,9,10,11] have demonstrated the benefits in terms of PFS and OS of ibrutinib administered continuously as single-agent or combined with anti-CD20 agents in both previously untreated and relapsed/refractory (R/R) settings, irrespective of the presence or absence of high-risk genomic abnormalities, such as *del(17p)*/*TP53* mutation [12]. These trials have also shown a tolerable safety profile across a broad patient population, including older and unfit patients, those with multiple comorbidities, and younger and fit patients. Extended treatment improved the depth of response, demonstrating the sustained clinical benefit and disease control associated with continuous ibrutinib treatment [13,14,15]. Recently, promising results for the combination of ibrutinib with venetoclax have also been suggested [16]. Currently, ibrutinib is the BTKi with the longest follow-up data in the first line in CLL/SLL, and the long-term results of RESONATE-2 reveal a 7-year PFS of 59% and an estimated 7-year OS of 78% [13].

It is well-known that patients enrolled in RTCs poorly reflect those seen in clinical practice, who typically are older, have higher burden of comorbidities, and have more unfavorable prognostic features [17]. Real-world data are essential to further substantiate the results of RCTs and to validate the effectiveness and safety of treatments beyond the confines of controlled trial settings.

Several observational studies have confirmed ibrutinib as a highly effective and generally well-tolerated drug when administered in routine clinical practice [18,19,20,21,22,23,24,25,26,27,28,29,30,31,32,33]. However, the real-world evidence is predominantly derived from retrospective studies conducted mostly on pretreated patients, including those treated in compassionate-use programs of ibrutinib [20,22] and single-institution patient cohorts [25,33,34]. In addition, the heterogeneity in clinical practice across countries makes national experiences of particular relevance.

The EVIdeNCE study (ClinicalTrials.gov Identifier: NCT03720561) is a prospective, multicenter, non-interventional investigation designed to describe ibrutinib utilization patterns in a real-world Italian setting [35]. The first interim analysis at 1 year after the start of the study indicated high ibrutinib persistence and no new relevant safety concerns [35]. In this report, we present the final results from the EVIdeNCE study over the 2-year clinical observation period. The primary objective was to evaluate in patients with CLL the retention of ibrutinib treatment at 2 years in routine clinical practice in Italy. Additionally, we described dose reductions, temporary interruptions and discontinuations, clinical effectiveness outcomes, and ibrutinib’s safety profile.

## 2. Materials and Methods

EVIdeNCE (NCT03720561) is an Italian, multicenter, observational, prospective cohort study on consecutive patients with CLL who started ibrutinib treatment per routine clinical practice in 39 hematological institutions [35]. Study enrollment took place from November 2018 to October 2019, a period when first-line ibrutinib reimbursement by the Italian National Health Service (NHS) was limited to patients with CLL <65 years of age with high-risk genomic features, patients aged 65–69 with at least one comorbidity, or elderly patients. The main inclusion criteria were as follows: patients aged ≥ 18 years, clinically active symptomatic CLL, either treatment-naïve (TN) or relapsed/refractory (R/R) according to the International Workshop on Chronic Lymphocytic Leukemia (iwCLL) criteria [36], and eligible for ibrutinib treatment reimbursement according to the Italian NHS. Exclusion criteria were participation in any experimental clinical trials; contraindications to ibrutinib use as described in the Summary of Product Characteristics (SmPC), treatment with any investigational compound or any invasive investigational medical device within 30 days before the start of ibrutinib treatment; and pregnant or breastfeeding women. A history of cardiovascular disease was not an exclusion criterion. Patients were followed for a 24-month period, regardless of whether they discontinued ibrutinib treatment. Follow-up visits were scheduled every 3 months during the first year and every 6 months thereafter. The study was conducted according to the Declaration of Helsinki and principles of good clinical practice (GCPs), with the approval of an Independent Ethics Committee. All patients provided written informed consent to participate in the study. Data collection was performed at baseline and during follow-up visits throughout the 24-month observation period. Demographic and clinical data were extracted mainly from medical records and entered into an electronic case report form (e-CRF). Additionally, participating physicians obtained patient-reported outcome (PRO) data from patients. At baseline, demographics, patient clinical characteristics, comorbidities, detailed medical history, previous CLL characteristics, and treatments were collected. Prospective data collection included dose modification, with reasons; treatment interruptions and permanent treatment discontinuation, including reasons; measures of effectiveness and treatment response according to the iwCLL 2018 guidelines [36]; hematologic and biochemistry parameters; levels of Ig types; and vital signs. Adverse events (AEs) were collected and classified according to the National Cancer Institute Common Terminology Criteria for adverse events (CTCAE) version 5.0. Concomitant therapies, medical resource utilization, and any subsequent non-ibrutinib therapy were also recorded. Furthermore, participants receiving ibrutinib treatment were requested to complete health-related quality-of-life questionnaires (EQ-5D-5L and EORTC QLQ-C30).

This report followed the “Strengthening the Reporting of Observational Studies in Epidemiology” (STROBE) guidelines for reporting observational studies.

### 2.1. Sample Size Determination

No formal confirmatory hypothesis testing or statistical power calculations were pre-specified for this observational descriptive study. A sample size of at least 300 patients was chosen for feasibility reasons.

### 2.2. Statistical Analysis

Patients were classified according to the line of ibrutinib administration as follows: first line (1L), second line (2L), or third-line or later (≥3L).

The primary endpoint was the 2-year ibrutinib retention rate, defined as the proportion of patients still on ibrutinib at that time point over the number of patients at risk. Treatment interruption was defined as not taking ibrutinib for ≤3 months, and treatment discontinuation was defined as not taking ibrutinib for >3 months or permanently. Additional endpoints included time to ibrutinib discontinuation (TTD), best overall response, PFS, OS, and safety. Each patient’s endpoint was assessed by site investigators.

The response assessment included complete response (CR), partial response (PR), or partial response with lymphocytosis (PRL) [36]. TTD was defined as the time from ibrutinib start to ibrutinib permanent discontinuation. PFS was defined as the time from ibrutinib start to disease progression or death from any cause. OS was defined as the time from ibrutinib start to death from any cause.

Continuous variables were presented as mean values ± standard deviations or median values (interquartile ranges, IQRs), and categorical variables were reported as numbers and percentages. The 95% confidence interval (CI) for the retention rate was calculated based on the Clopper–Pearson exact method for the binomial proportion. Survival curves for TTD, PFS, and OS were estimated using the Kaplan–Meier method. The 1- and 2-year probability of surviving were also calculated.

## 3. Results

### 3.1. Study Population and Baseline Characteristics

Out of the 311 patients with CLL enrolled in the EVIdeNCE study, 1 did not start ibrutinib, and another was excluded for participating in a separate clinical trial. This resulted in 309 eligible patients for analysis. Among the total participants, 118 (38.2%) were TN at baseline and initiated ibrutinib treatment in the 1L, while 191 (61.8%) had R/R disease and commenced ibrutinib treatment in the 2L (*n* = 127, 41.1%) or in the third line or beyond (≥3L) (*n* = 64, 20.7%). The median time from CLL diagnosis to the initiation of ibrutinib therapy was 1.7 years (interquartile range, IQR: 0.7–4.3) in the 1L group, 6.4 years (IQR: 4.6–9.0) in the 2L group, and 8.8 years (IQR: 6.4–12.4) in the ≥3L group.

A total of 229 patients completed the 24-month observational period, while 80 (25.9%) discontinued prematurely: 41 patients died, 11 withdrew the informed consent/privacy form, 24 were lost to follow-up or missed the final follow-up visit, and 4 discontinued for other reasons. The median duration of patient follow-up was 23.9 months (IQR: 22.5–24.4).

At the start of ibrutinib treatment, no significant differences were observed across the various treatment lines. The median age of patients was 72 years in the 1L cohort and 71 years in the 2L and ≥3L cohorts, and the percentage of male patients was 63%, 61%, and 67%, respectively (Table 1). Overall, 195 patients (63.1%) had at least one clinically relevant comorbidity: 31 (10.0%) patients had a prior malignancy, 31 (10.0%) had prior hepatitis B or C infection, 26 (8.4%) had diabetes, and 17 (5.5%) had a significant respiratory disease. Among 103 (33.3%) patients with a history of cardiovascular disorders, 79 (76.7%) had ongoing cardiovascular disorders at the start of ibrutinib therapy. The large majority of patients had an ECOG-PS of 0–1, with no difference in the rate of CIRS score > 6 across the different lines of therapy (*p* = 0.240). In total, 54 (48.2%) patients in 1L, 64 (52.0%) in 2L, and 34 (59.6%) in ≥3L were classified as Rai stage III-IV (*p* = 0.464). Moreover, *del(17p)* or *TP53* mutation was found in 32/60 (53.3%) tested patients who received 1L treatment, 23/55 (41.8%) tested patients who received 2L, and 14/23 (60.9%) tested patients who received ≥3L treatment (*p* = 0.243). Unmutated *IGHV* was reported in 38/56 (67.9%), 39/58 (67.2%), and 17/21 (81.0%) patients, treated in 1L, 2L, and ≥3L, respectively (*p* = 0.469).

Among patients who received ibrutinib as 2L therapy, bendamustine-R (BR) (*n* = 40 or 31.5%) and fludarabine–cyclophosphamide–rituximab (FCR) (*n* = 32 or 25.6%) were the most frequent chemo-immunotherapies (CITs) previously utilized. Among patients who received ibrutinib as ≥3L therapy, the most common CIT immediately preceding ibrutinib was BR (*n* = 26 or 40.6%) or R-chlorambucil (*n* = 7, 10.9%).

### 3.2. Ibrutinib Starting Dose and Concomitant Medications

At the initiation of the study, in the overall sample, 231 patients (74.8%) received the recommended daily dose of 420 mg (Supplementary Table S1); the proportion was consistent across treatment line groups (1L, 72.0%; 2L, 79.5%; and ≥3L, 70.3). Thirty-nine patients (12.6%) started treatment at a dose of 140 mg, and another thirty-nine patients (12.3%) began with 280 mg. Among the 78 patients who initially started with reduced doses of ibrutinib, 53 (67.9%) eventually escalated their dose to 420 mg daily (Supplementary Table S2). With the exception of one patient, all received single-agent ibrutinib. In total, 35% of patients received concomitant antihypertensive drugs (mainly beta-blockers, alpha-blockers, and ACE inhibitors), 34% received inhibitors of uric acid, 13% received proton pump inhibitors, and 7% received platelet anti-aggregates. Reflecting the policy of different hematological institutions, 31% of patients received prophylaxis for pneumocystis pneumonia with trimethoprim–sulfamethoxazole, and 23% received an antiviral prophylaxis.

### 3.3. Retention and Discontinuation Rate

**Figure 1:**
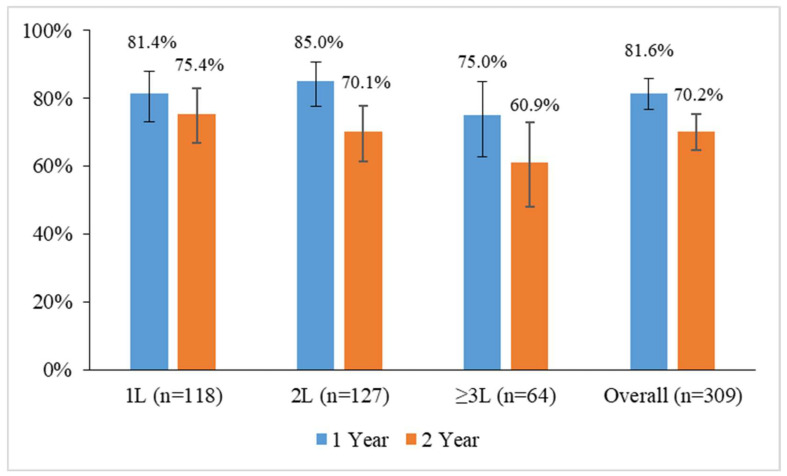
Ibrutinib retention rates at 1 and 2 years in patients with chronic lymphocytic leukemia by the line of treatment in which the drug was administered in the prospective real-world EVIdeNCE study. Vertical lines represent 95% confidence intervals.

The overall retention rate for 2 years was 70.2% (95% CI: 64.8–75.3%, *n* = 217). The retention rate was higher for patients who received ibrutinib as 1L (75.4%; 95% CI: 66.7–82.9%, *n* = 89) in comparison to those who were given ibrutinib as 2L (70.1%; 95% CI: 61.3–77.9%; *n* = 89) or ≥3L (60.9%; 95% CI: 47.9–72.9%, *n* = 39) (Figure 1).

**Figure 2:**
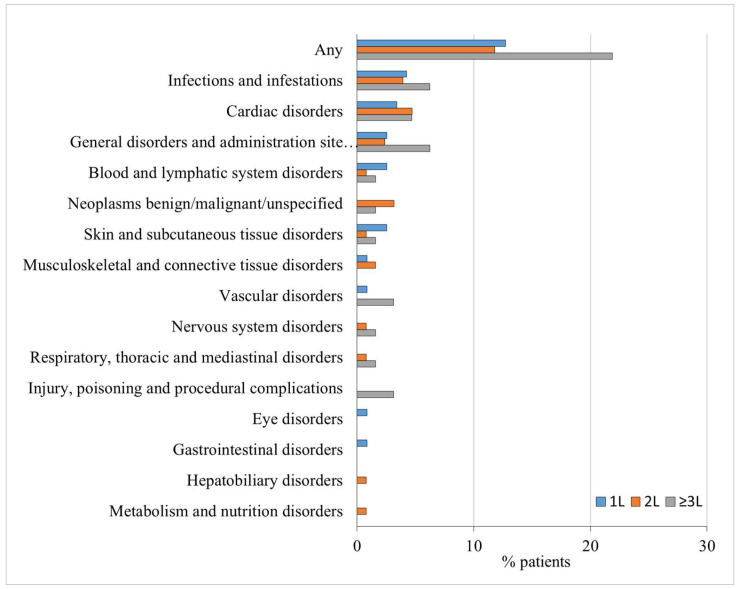
Adverse events of any grade leading to ibrutinib discontinuation by the line of treatment in which the drug was administered in the prospective real-world EVIdeNCE study.

A temporary interruption of ibrutinib treatment occurred in 107 patients (34.6%), with a median interruption duration of 2 weeks (Table 2). The most common reason for this interruption was an AE. The majority of patients (*n* = 63, 60%) experienced a single treatment interruption period. In total, 92 patients (29.8%) experienced a permanent discontinuation of ibrutinib. The discontinuation of ibrutinib showed no correlation with its use as a first-line (1L) or later-line therapy. Among the patients, 29 (24.6%) were in the 1L group, 38 (29.9%) were in the second-line (2L) group, and 25 (39.1%) were in the third-line or later (≥3L) treatment. The primary reasons for discontinuing permanently ibrutinib were, in order, AEs (44 cases, 14.2%), death (18 cases, 5.8%), and progressive disease (15 cases, 4.9%). The most common AE leading to ibrutinib discontinuation was infection in patients who received ibrutinib as 1L (*n* = 5, 4.2%) or ≥3L therapy (*n* = 4, 6.3%), while permanent discontinuation of ibrutinib was primarily due to cardiovascular complications in patients who received ibrutinib as 2L treatment (*n* = 6, 4.7%) (Figure 2).

The estimated persistence rates appear higher for 1L patients (Supplementary Figure S1A) and in patients younger than 70 years compared to older individuals (Supplementary Figure S1B). Following the discontinuation of ibrutinib, 29 (9.4%) patients started a new CLL therapy (1L, 9.3%; 2L, 8.7%; and 3L, 10.9%) (Table 2). The most frequently administrated subsequent therapy was venetoclax (52%).

### 3.4. Clinical Response and Survival Outcomes

A response to ibrutinib was achieved by 202 (75.9%) patients (1L, 80.8%; 2L, 75.2%; and 3L, 68.4%) (Table 3). In particular, a clinical CR was achieved by 18.4% of patients (1L, 27.8%; 2L, 16.2%; and ≥3L, 5.3%).

During the observation period, 64 (20.7%) patients experienced a disease progression (1L, *n* = 17; 2L, *n* = 28; ≥3L, *n* = 19), and 41 patients died (1L, *n* = 10; 2L, *n* = 19; ≥3L, *n* = 12). The causes of death included infections (*n* = 19), disease progression (*n* = 9), second malignancies (*n* = 5), and cardiovascular events (*n* = 3).

The median PFS was not reached, and the 2-year PFS across treatment lines was 85.4% in 1L, 80.0% in 2L, and 70.1% in ≥3L (Table 3 and Supplementary Figure S2). The 2-year OS rates were 91.7% in 1L, 86.2% in 2L, and 80.0% in ≥3L patients.

In analyses by starting dose, the overall response was 79% among patients starting full dose ibrutinib and 67% among those starting reduced doses; the corresponding 2-year PFSs were 82.8% and 71.8%, respectively.

A prior history of cardiovascular disease or subsequent cardiovascular events during ibrutinib did not impact patients’ clinical outcomes, with a 1- and 2-year PFS of 89.0% and 82.3%, respectively, in patients with cardiovascular diseases.

### 3.5. Safety

During the observation period, 233 (75.4%) patients had at least one AE, and 107 (34.6%) had at least one grade 3–4 AE. The most common AEs of any grade were infections (30.7%), bleeding (12.9%), fatigue (10.0%), neutropenia (9.7%), diarrhea (9.1%), and atrial fibrillation (8.1%) (Table 4); 9.1% of patients developed a second malignancy. Grade 3–4 events were relatively rare, with neutropenia occurring in 26 (8.4%) patients, infection in 20 (6.5%), and atrial fibrillation in 12 (3.9%). One sudden death was registered. The cumulative risk of developing infections, cardiovascular events, and atrial fibrillation was similar in TN and R/R patients (Supplementary Figure S3). Notably, the cumulative risk of developing infections and cardiovascular disorders gradually increased over the study period, while for atrial fibrillation, such a risk plateaued after the first 6 months of ibrutinib therapy.

Among the 79 patients with cardiovascular disorders ongoing at baseline, 11 (13.9%) developed a subsequent ibrutinib-related cardiovascular event.

## 4. Discussion

Herein, we present the final results of the EVIdeNCE study, the largest prospective investigation in Italy enrolling patients with CLL who received ibrutinib monotherapy across multiple lines of treatment in real-world clinical settings. The study highlights a noteworthy prevalence of patients exhibiting comorbidities, notably cardiovascular disorders and unfavorable genetic features. These findings align with the reimbursement criteria for ibrutinib treatment in Italy during the study period. Nevertheless, the survival outcomes of real-world patients included in this study were slightly inferior compared to the PFS and OS reported in clinical trials involving single-agent ibrutinib. For instance, in the RESONATE-2 trial, TN patients achieved a 2-year PFS of 89% [12], while R/R patients in the RESONATE trial had a 2-year PFS rate of approximately 75% [15]. Nevertheless, it is important to highlight that the proportion of R/R patients with only one prior treatment was 66% in the EVIdeNCE study, contrasting with the 18% reported in the RESONATE trial [15].

When considering the genetic profile of patients, the retrospective real-world Canadian cohort by Khelifi et al. [28] reported a 2-year OS of 83.9% in a CLL population with a similar high proportion of patients with adverse genetic features. Moreover, a nationwide Italian analysis based on an administrative dataset from the Italian Medicines Agency (AIFA), including more than 740 patients with CLL with aberrant TP53 treated front-line with ibrutinib, showed 2-year treatment persistence and OS rates of 63% and 83%, respectively [37].

In line with data from clinical trials [8,12,38] and real-world retrospective studies [31,37,39], this prospective real-world study shows a high rate of patients with CLL still on ibrutinib at 2 years. Discontinuation rates vary across real-world studies on patients with CLL treated with ibrutinib, from around 15% over a median follow-up of 3 months in a French cohort (97% R/R patients) [22] to 65% over a median follow-up of 25 months in a study among elderly Medicare beneficiaries [40]. Differences in the baseline clinical and biological characteristics of patients, number of prior treatments, years of treatment, patient management, and heterogeneity in the follow-up periods may contribute to such a wide variation. Interestingly, our discontinuation rate is similar to the average discontinuation rate reported in long-term clinical trials with ibrutinib [41]. Given the higher risk of refractory CLL, AEs, and cytopenia in patients receiving ibrutinib as advanced-line therapy after prior chemoimmunotherapy, the 2-year retention rate was higher in patients receiving ibrutinib in the front line (75%) compared to that in later lines (2L, 70%; ≥3L, 61%). Interestingly, a real-world multicentric German study (REALITY study) reported higher adherence and retention rates in patients with high, compared with those with low, acceptance of the disease, suggesting emotive support for patients with lower acceptance of CLL as a possible strategy to improve the compliance and duration of ibrutinib treatment [42].

The most common reason for treatment discontinuation was AE/toxicity. This observation is in line with the majority of real-world studies that identified AEs as the main reason for discontinuing ibrutinib [18,24,26,27,28,29,41] and possibly other BTKi [43]. Conversely, disease progression was the most frequent cause of treatment discontinuation recorded in RTCs. The difference in the primary reason for discontinuing ibrutinib between controlled trials and real-world studies can be attributed to the fact that the latter typically involve older patients with lower performance statuses who receive less intensive monitoring. Infections in the 1L and ≥3L cohorts and cardiac disorders in the in 2L were the most frequent AEs leading to treatment discontinuation.

The AE pattern observed in EVIdeNCE aligns with the AE profile documented in clinical trials of ibrutinib. Notably, infections, bleeding issues, fatigue, neutropenia, and diarrhea emerge as the predominant types of AEs. The prevalence of atrial fibrillation among patients was 8%, and it increased with each treatment line: 1L, 6.8%; 2L, 8.7%; and ≥3L, 9.4%. In the RESONATE-2 including TN patients, with a median follow-up of 29 months, atrial fibrillation occurred in 10% of patients [12]. In the RESONATE trial, with more than 41 months of treatment, the rate of patients with atrial fibrillation in R/R patients was 22% [15]. Interestingly, we found that the cumulative incidence of AEs of atrial fibrillation gradually increased, approaching a plateau beyond 6 months, indicating the first 6-month period of treatment as more critical for atrial fibrillation events. We recorded a lower rate of patients with hypertension, 4.2%, than in RESONATE-2 and RESONATE trials (20%). This observation may reflect, at least in part, a non-systematic reporting of hypertension in clinical practice. It has been hypothesized that ibrutinib is associated with ventricular arrhythmias [44]. No ventricular arrhythmias were reported; however, one case of sudden cardiac death was recorded in our study. Notably, in the present study, 33% of patients had pre-existing cardiovascular diseases, and 14% developed a cardiovascular event during the study. The clinical outcomes of patients with a prior or concomitant cardiovascular AE in terms of PFS were comparable to those of overall patients, with a 2-year PFS of about 80%. This observation suggests that optimal co-management of both cardiovascular disorders and ibrutinib therapy had a favorable impact on clinical outcomes.

We postulate that the higher rate of temporary interruptions observed in the 1L cohort when compared to the advanced lines cohorts may be attributed to a heightened awareness of the toxic effects among TN patients and an increased concern about the potential risks associated with refractory disease in the R/R setting.

The extent of dose reductions (25%) and dose interruptions appears much higher in real-world investigations than in clinical trials due to more stringent rules for dose modifications/treatment interruptions [41,45]. Some studies indicate that ibrutinib interruptions might affect clinical outcomes [18,45], but not dose reductions [20,45,46], pointing to the importance of continuous therapy [47]. Recent real-world findings suggest that dose flexibility can be an effective strategy to manage AEs and maintain long-term treatment [48,49,50]. In a pooled analysis of seven clinical trials, including over 1200 patients, ibrutinib dose reductions after early cardiac AEs did not impact PFS or OS [49]. These data and our findings suggest that dose flexibility is an effective treatment approach to optimize outcomes, including in patients who develop cardiovascular events [51].

The major strength of our study is the prospective design with the enrollment of a relatively large number of consecutive patients from several Italian hematological sites who required ibrutinib therapy in clinical practice. This study’s design limited selection bias. In addition, data were collected in a dedicated e-CRF. However, some limitations of this study should be mentioned, such as the relatively short follow-up and the lack of genetic data in a large number of patients, reflecting the low rate of genetic testing in the Italian clinical practice.

## 5. Conclusions

The EVIdeNCE prospective study provides a unique perspective on the clinical course of patients with CLL treated with single-agent ibrutinib in Italian clinical practice. In this unselected CLL patient population, characterized by a high level of comorbidities and unfavorable prognostic factors, the 2-year persistence rate was relatively high, and survival outcomes were favorable. As anticipated, patients treated upfront with ibrutinib exhibited more favorable outcomes, reaffirming the heightened efficacy of ibrutinib as an initial treatment for CLL. The safety profile of ibrutinib treatment was in line with that reported in selected patients included in clinical trials, with a relatively low rate of atrial fibrillation and hypertension. Of note, in this study, survival outcomes did not appear to be adversely impacted by pre-existing or concomitant cardiovascular disorders.

Taken together, the results of this study suggest that a better knowledge and expertise in managing AEs improved the long-term outcomes of patients with CLL treated with ibrutinib. This study included patients with characteristics typically seen in individuals with this type of leukemia. A significant number of patients continued taking ibrutinib for more than one year. These encouraging findings indicate that ibrutinib is a viable treatment for patients diagnosed with CLL. Furthermore, the reassuring safety profile and the possibility of dose reduction and flexibility associated with ibrutinib suggest a favorable benefit–risk profile for the novel ibrutinib–venetoclax combination [52,53,54]. The decision to use continuous therapy with ibrutinib as a single agent or in combination with venetoclax should be based on the patient’s clinical and biological characteristics, as well as the desired treatment outcome. In any case, new data on the issue from the real world are warranted.

## Figures and Tables

**Table 1:** Baseline demographic and clinical characteristics of patients with chronic lymphocytic leukemia initiated on ibrutinib by the line of treatment in which the drug was administered in the prospective real-world EVIdeNCE study.

Baseline Characteristics	Overall (*n* = 309) *n* (%)	1L (*n* = 118) *n* ^ (%)	2L (*n* = 127) *n* ^ (%)	≥3L (*n* = 64) *n* ^ (%)	*p*-Value ^¶^
Age at ibrutinib initiation					
<65	71 (23.0)	18 (15.3)	38 (29.9)	15 (23.4)	0.088
65–69	52 (16.8)	24 (20.3)	17 (13.4)	11 (17.2)	
≥70	186 (60.2)	76 (64.4)	72 (56.7)	38 (59.4)	
Median (IQR)	71 (65–77)	72 (67–77)	71 (63–74)	71 (65–78)	
Male sex	195 (63.1)	74 (62.7)	78 (61.4)	43 (67.2)	0.733
ECOG-PS					
0–1	238 (90.2)	82 (89.1)	109 (92.4)	47 (87.0)	0.508
≥2	26 (9.8)	10 (10.9)	9 (7.6)	7 (13.0)	
Unknown	45	26	9	10	
CIRS score					
<6	174 (71.9)	62 (66.7)	76 (77.6)	36 (70.6)	0.240
≥6	68 (28.1)	31 (33.3)	22 (22.4)	15 (29.4)	
Unknown	67	25	29	13	
History of significant CVD *	103 (33.3)	45 (38.1)	37 (29.1)	21 (32.8)	0.326
Rai Staging System at ibrutinib initiation					
Stage 0	12 (4.1)	3 (2.7)	6 (4.9)	3 (5.3)	0.464
Stage I-II	128 (43.8)	55 (49.1)	53 (43.1)	20 (35.1)	
Stage III-IV	152 (52.0)	54 (48.2)	64 (52.0)	34 (59.6)	
Unknown	17	6	4	7	
Mutational status ^¥^					
Unmutated *IGHV*	94/135 (69.6)	38/56 (67.9)	39/58 (67.2)	17/21 (81.0)	0.469
*TP53* mutation	52/164 (31.7)	25/68 (36.8)	15/65 (23.1)	12/31 (38.7)	0.154
Any cytogenetic alterations	181/266 (68.0)	67/102 (65.7)	77/113 (68.1)	37/51 (72.5)	0.692
*Del11q*	47/266 (17.7)	12/102 (11.8)	25/113 (22.1)	10/51 (19.6)	0.128
*Del17p*	56/266 (21.1)	28/102 (27.5)	19/113 (16.8)	9/51 (17.6)	0.129
*Del17p* or *TP53* mutation	69/138 (50.0)	32/60 (53.3)	23/55 (41.8)	14/23 (60.9)	0.243

CIRS, Cumulative Illness Rating Scale; ECOG-PS, Eastern Cooperative Oncology Group performance status; CVD, cardiovascular disease; IGHV, immunoglobulin heavy-chain variable region gene; IQR, interquartile range. ^ Values are represented as absolute numbers and percentages unless otherwise specified. * Included uncontrolled or symptomatic arrhythmias, atrial fibrillation, congestive heart failure, myocardial infarction, and hypertension. ^¥^ Percentages are calculated over the number of patients with genetic test performed. ^¶^ From chi-square test.

**Table 2:** Treatment interruptions, discontinuations, and subsequent therapies in patients with chronic lymphocytic leukemia (CLL) treated with ibrutinib and subsequent CLL therapies by the line of treatment in which the drug was administered in the prospective real-world EVIdeNCE study.

Event Type Description	1L (*n* = 118) *n* (%)	2L (*n* = 127) *n* (%)	≥3L (*n* = 64) *n* (%)	Overall (*n* = 309)
At least one temporary treatment interruption *	47 (39.8)	41 (32.3)	19 (29.7)	107 (34.6)
Treatment permanent discontinuation ^	29 (24.6)	38 (29.9)	25 (39.1)	92 (29.8)
Reason for discontinuation ^¶^				
AEs	15 (12.7)	15 (11.8)	14 (21.9)	44 (14.2)
Death	5 (4.2)	7 (5.5)	6 (9.4)	18 (5.8)
Disease progression	3 (2.5)	9 (7.1)	3 (4.7)	15 (4.9)
Second malignancy	1 (0.8)	5 (3.9)	2 (3.1)	8 (2.6)
Clinician’s choice	5 (4.2)	6 (4.7)	0 (0.0)	11 (3.6)
Other reason(s)	2 (1.7)	4 (3.1)	1 (1.6)	7 (2.3)
Time (months) to ibrutinib treatment discontinuation ˜, median (IQR)	5.7 (3.6–11.7)	11.3 (5.1–16.7)	7.2 (4.3–14.1)	6.9 (4.3–15.2)
Patients with subsequent CLL therapy	11 (9.3)	11 (8.7)	7 (10.9)	29 (9.4)

* ≤3 months without therapy. ^ >3 months without therapy. ^¶^ Multiple reasons could be indicated. ˜ Among patients who discontinued ibrutinib.

**Table 3:** Best treatment response according to physician evaluation, progression-free survival (PFS), and overall survival (OS) in patients with chronic lymphocytic leukemia treated with ibrutinib by the line of treatment in which the drug was administered in the prospective real-world EVIdeNCE study.

Efficacy Outcomes	1L (*n* = 118) *n* (%)	2L (*n* = 127) *n* (%)	≥3L (*n* = 64) *n* (%)	Overall (*n* = 309)
Best response				
ORR ^	84 (80.8)	79 (75.2)	39 (68.4)	202 (75.9)
CR *	29 (27.8)	17 (16.2)	3 (5.3)	49 (18.4)
PR	53 (51.0)	56 (53.3)	29 (50.9)	138 (51.9)
PR-L	2 (1.9)	6 (5.7)	7 (12.3)	15 (5.6)
SD	17 (16.3)	19 (18.1)	17 (29.8)	53 (19.9)
DP	3 (2.9)	7 (6.7)	1 (1.8)	11 (4.1)
Unknown	14	22	7	43
2-year PFS, %	85.4	80.0	70.1	79.3
2-year OS, %	91.7	86.2	80.8	85.6

ORR, overall response rate; CR, complete response; PR, partial response; PR-L, partial response with lymphocytosis; SD, stable disease; DP, Disease progression; 1L, first line; 2L, second line; ≥3L, third line or later. ^ According to iwCLL criteria. * Evaluated according to lymph nodes, liver/spleen, constitutional symptoms, and circulating lymphocyte count examination.

**Table 4:** Most frequent adverse events in patients treated with ibrutinib in total by the line of treatment in which ibrutinib was administered in the prospective real-world EVIdeNCE study *.

	1L (*n* = 118) *n* (%)	2L (*n* = 127) *n* (%)	≥3L (*n* = 64) *n* (%)	Overall (*n* = 309)
Any grade ^				
Any AE	88 (74.6)	93 (73.2)	52 (81.3)	233 (75.4)
Infection (including COVID-19)	35 (29.7)	37 (29.1)	23 (35.9)	95 (30.7)
Bleeding	16 (13.6)	13 (10.2)	11 (17.2)	40 (12.9)
Fatigue	9 (7.6)	14 (11.0)	8 (12.5)	31 (10.0)
Neutropenia	9 (7.6)	16 (12.6)	5 (7.8)	30 (9.7)
Diarrhea	12 (10.2)	10 (7.9)	6 (9.4)	28 (9.1)
Atrial fibrillation	8 (6.8)	11 (8.7)	6 (9.4)	25 (8.1)
Pyrexia	7 (5.9)	10 (7.9)	7 (10.9)	24 (7.8)
Arthralgia	8 (6.8)	10 (7.9)	2 (3.1)	20 (6.5)
Rash	9 (7.6)	8 (6.3)	3 (4.7)	20 (6.5)
Anemia	11 (9.3)	5 (3.9)	3 (4.7)	19 (6.1)
Hematoma	7 (5.9)	8 (6.3)	3 (4.7)	18 (5.8)
Muscle spasms	7 (5.9)	4 (3.1)	3 (4.7)	14 (4.5)
Hypertension	3 (2.5)	4 (3.1)	6 (9.4)	13 (4.2)
Back pain	5 (4.2)	4 (3.1)	2 (3.1)	11 (3.6)
Thrombocytopenia	1 (0.8)	7 (5.5)	3 (4.7)	11 (3.6)
Grade 3–4 ˜				
Any AE	33 (28.0)	44 (34.6)	20 (31.3)	107 (34.6)
Neutropenia	9 (7.6)	12 (9.4)	5 (7.8)	26 (8.4)
Infection	9 (7.6)	7 (5.5)	6 (9.4)	20 (6.5)
Atrial fibrillation	3 (2.5)	5 (3.9)	4 (6.3)	12 (3.9)
Anemia	5 (4.2)	1 (0.8)	2 (3.1)	8 (2.6)
Hypertension	0 (0.0)	2 (1.6)	3 (4.7)	5 (1.6)
Arthralgia	2 (1.7)	3 (2.4)	0 (0.0)	5 (1.6)
Lymphocytosis	2 (1.7)	2 (1.6)	1 (1.6)	5 (1.6)

* Events that occurred after ibrutinib discontinuation were also considered. ^ Occurring in at least 10 patients. ˜ Occurring in at least 5 patients.

## Data Availability

The datasets generated and/or analyzed during the current study are available from the corresponding author upon reasonable request. Requests for the data underlying this publication require a detailed, hypothesis-driven statistical analysis plan that is collaboratively developed by the requestor and company subject matter experts.

## Supplementary Materials

The following supporting information can be downloaded at https://www.mdpi.com/article/10.3390/cancers16061228/s1, Table S1: Details on first dose of ibrutinib in patients with chronic lymphocytic leukemia in EVIdeNCE study; Table S2: Details on ibrutinib dose modification during treatment in patients with chronic lymphocytic leukemia in EVIdeNCE study; Figure S1: Kaplan–Meier curves for the time to ibrutinib permanent discontinuation during the 24-month observational period by the line of treatment in which the drug was administered (Panel A) and by patients’ age (Panel B) in the prospective real-world EVIdeNCE study; Figure S2: Kaplan–Meier progression free survival (Panel A) and overall survival (Panel B) curves by the line of treatment in which the drug was administered in the prospective real-world EVIdeNCE study; Figure S3: Cumulative incidence of cardiovascular adverse events (AEs), atrial fibrillation, and infections in treatment-naïve (Panel A) and relapsed/refractory (Panel B) patients with chronic lymphocytic leukemia treated with ibrutinib in the prospective real-world EVIdeNCE study.
