# Supplementary material for: Real-World Outcome of Treatment with Single-Agent Ibrutinib in Italian Patients with Chronic Lymphocytic Leukemia: Final Results of the EVIdeNCE Study
Source: Cancers (Basel). 2024 Mar 20;16(6):1228. doi: 10.3390/cancers16061228 (PMC10969011; doi:10.3390/cancers16061228)
Supplement: Supplementary file 1 [file cancers-16-01228-s001.zip › cancers-2859395-supplementary.pdf]

Real-World Outcome of Treatment with Single-Agent Ibrutinib in Italian Patients with Chronic Lymphocytic Leukemia: Final Results of the EVIdeNCE Study

Supplementary materials

Figure S1. Kaplan-Meier curves for the time to ibrutinib permanent discontinuation during the 24-month observational period by the line of treatment in which the drug was administered (Panel A) and by patients' age (Panel B) in the prospective real-world EVIdeNCE study

Panel A

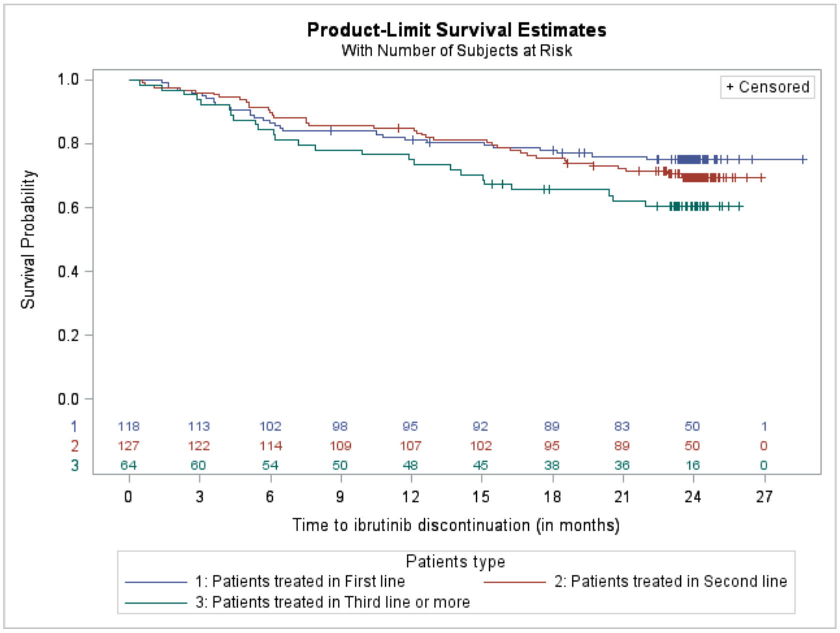

Panel B

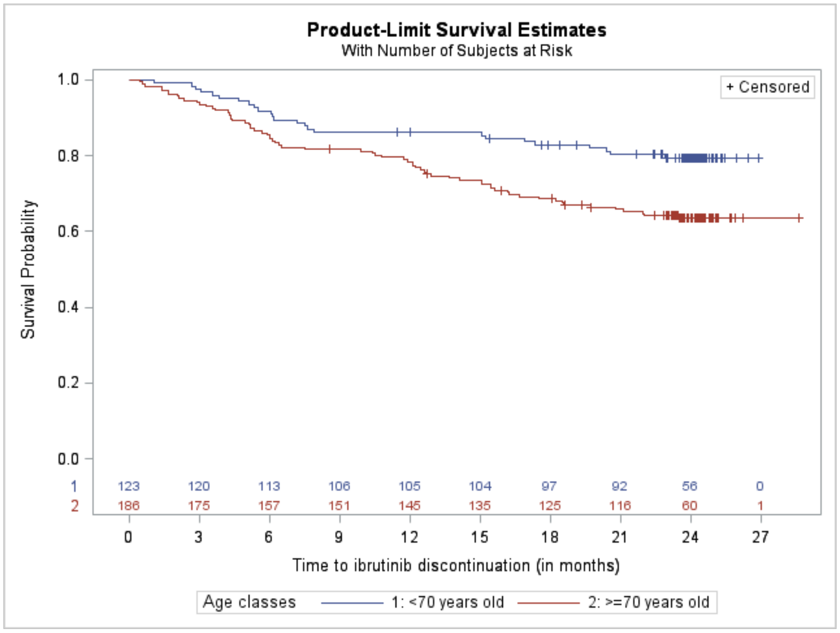

Figure S2. Kaplan-Meier progression free survival (Panel A) and overall survival (Panel B) curves by the line of treatment in which the drug was administered in the prospective real-world EVIdeNCE study

Panel A

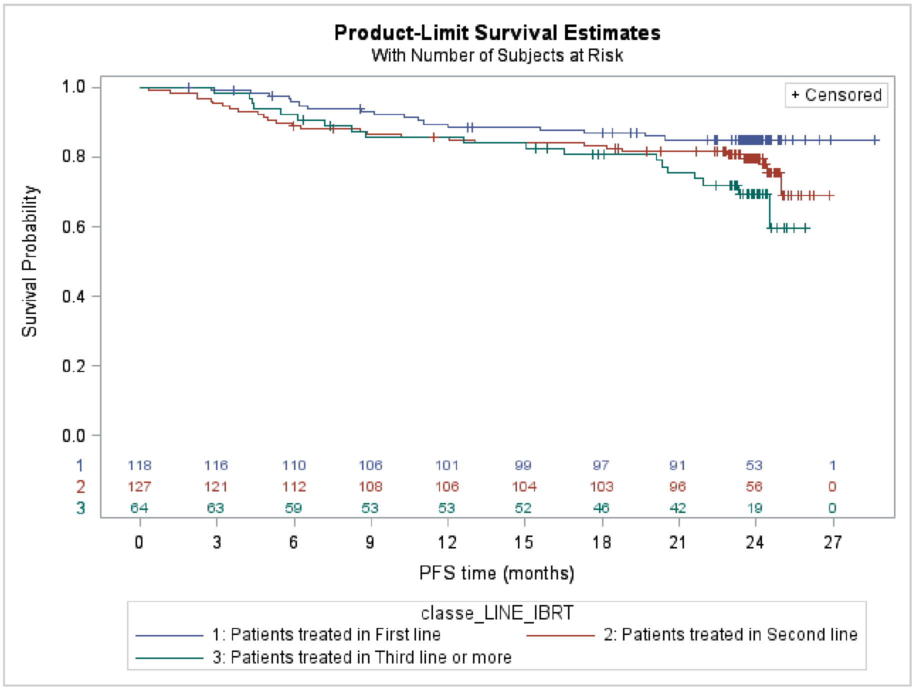

Panel B

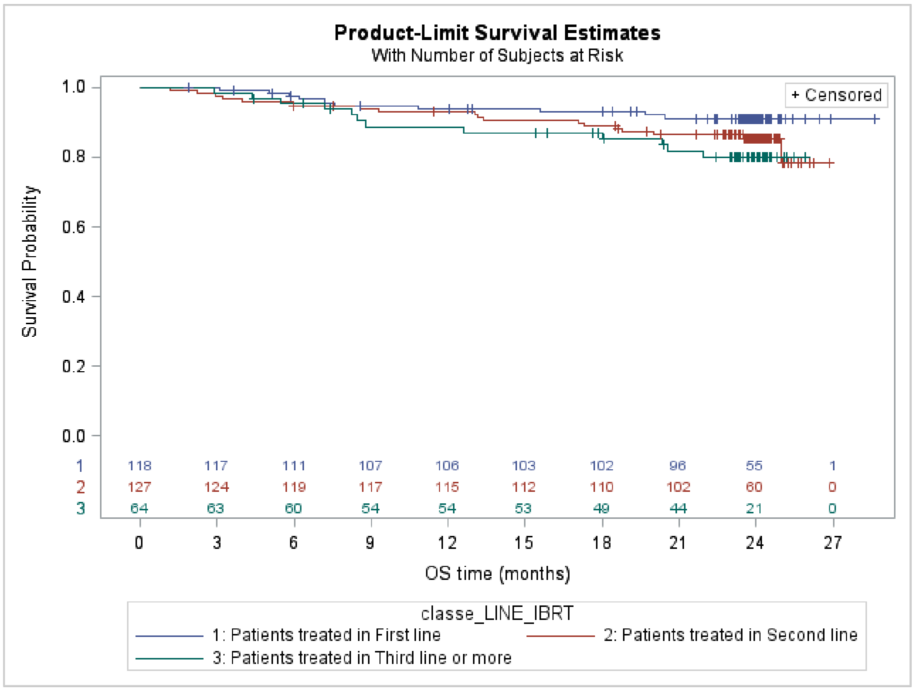

Figure S3. Cumulative incidence of cardiovascular adverse events (AEs), atrial fibrillation, and infections in treatment-naïve (Panel A) and relapsed/refractory (Panel B) patients with chronic lymphocytic leukemia treated with ibrutinib in the prospective real-world EVIdeNCE study

Panel A

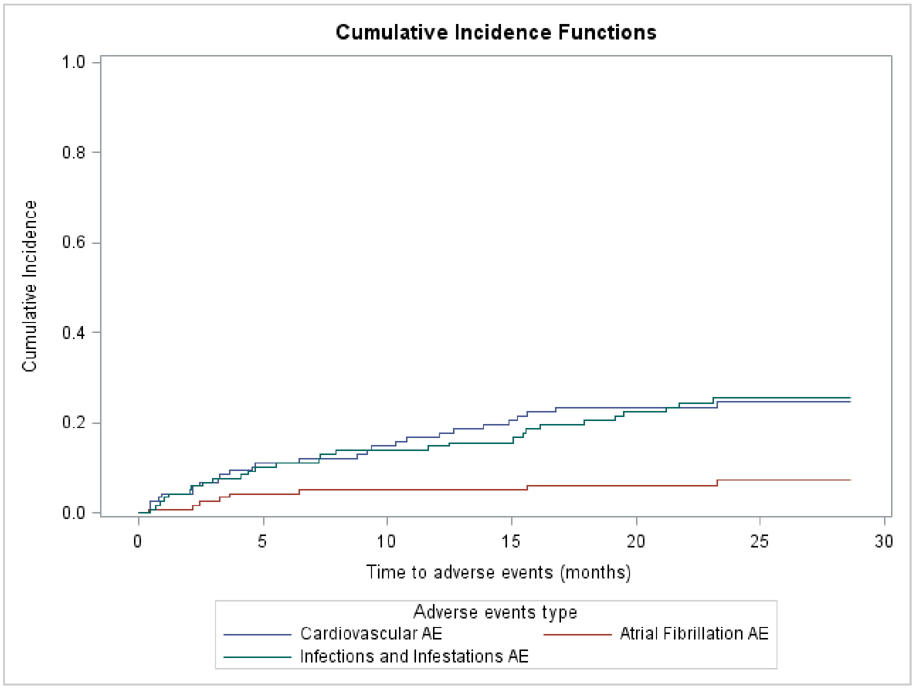

Panel B

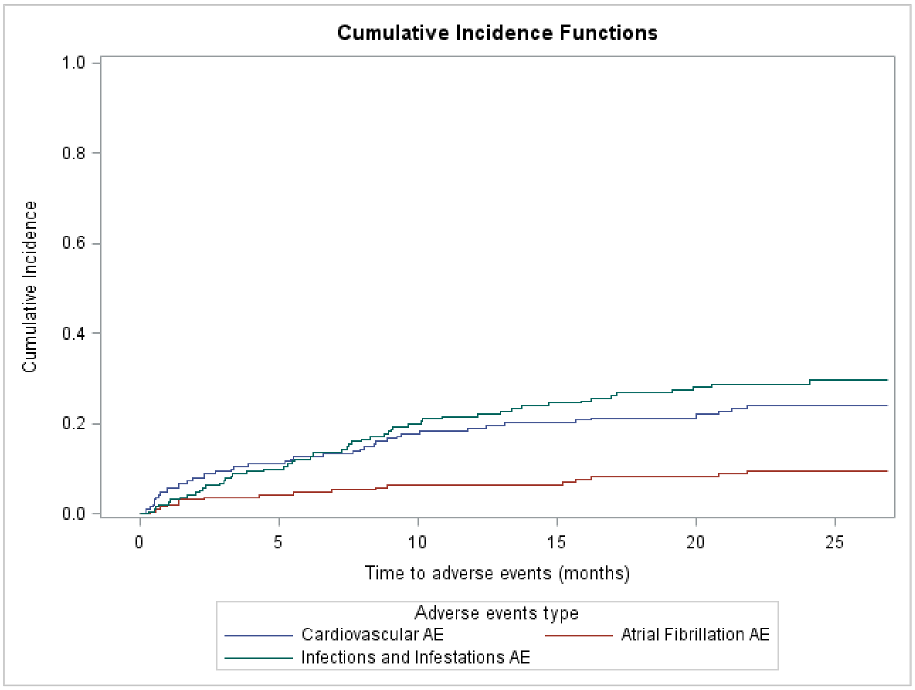

Table S1. Details on first dose of ibrutinib in patients with chronic lymphocytic leukemia in EVIdeNCE Study.

	1L (N=118) n (%)	2L (N=127) n (%)	≥3L (N=64) n (%)	Overall (N=309)
Posology of first dose of ibrutinib				
420 mg once daily (ibrutinib only)	85 (72.0)	101 (79.5)	45 (70.3)	231 (74.8)
140 mg once daily (ibrutinib only)	19 (16.1)	12 (9.4)	8 (12.5)	39 (12.6)
280 mg once daily (ibrutinib only)	14 (11.9)	13 (10.2)	11 (17.2)	38 (12.3)
280 mg once daily (ibrutinib in combination)	0 (0.0)	1 (0.8)	0 (0.0)	1 (0.3)

Table S2. Details on ibrutinib dose modification during treatment in patients with chronic lymphocytic leukemia in EVIdeNCE Study.

	1L (N=118) n (%)	2L (N=127) n (%)	≥3L (N=64) n (%)	Overall (N=309)
Patients receiving full dose (420 mg once daily) of ibrutinib during the observation period at any time	106 (89.8)	122 (96.1)	56 (87.5)	284 (91.9)
Patients with ibrutinib first dose equal to 420 mg once daily with at least a dose decrease^	20 (23.5)	24 (23.8)	15 (33.3)	59 (25.5)
Patients with ibrutinib first dose less than 420 mg once daily increasing dose to 420 mg¶	21 (63.6)	21 (80.8)	11 (57.9)	53 (67.9)

^ Percentages were calculated over the number of patients with starting dose equal to 420 mg once daily.

¶ Percentages were calculated over the number of patients with starting dose less than 420 mg once daily.
